# Factors associated with knee pain in 5148 women aged 50 years and older: A population-based study

**DOI:** 10.1371/journal.pone.0192478

**Published:** 2018-03-08

**Authors:** Kyoung Min Lee, Seung-Baik Kang, Chin Youb Chung, Moon Seok Park, Dong-wan Kang, Chong bum Chang

**Affiliations:** 1 Department of Orthopaedic Surgery, Seoul National University Bundang Hospital, Gyeonggi, South Korea; 2 Department of Orthopaedic Surgery, SMG‑SNU Boramae Medical Center, Seoul National University College of Medicine, Seoul, South Korea; University of Umeå, SWEDEN

## Abstract

**Objective:**

This study was performed to investigate the factors associated with the level of knee pain in a nationally representative sample of noninstitutionalized women aged 50 years or older.

**Methods:**

Women aged 50 years or older were selected and included in the data analyses from the Korean National Health and Nutrition Examination Surveys (2010–2013). Those having malignant diseases or using osteoarthritis medication were excluded. Significant factors associated with the level of knee pain were analyzed using multivariate regression analysis.

**Results:**

A total of 5148 women (average age, 62.9 years; standard deviation, 9.3 years) were included. For women without knee osteoarthritis, level of hip pain (p<0.001), presence of back pain (p<0.001), age (p<0.001), and body mass index (BMI) (p<0.001) were found to be significant factors associated with the level of knee pain. For women with knee osteoarthritis, the radiographic grade of knee osteoarthritis (p<0.001), presence of back pain (p<0.001), level of hip pain (p<0.001), presence of depressive symptoms (p<0.001), and BMI (p = 0.026) were the factors significantly associated with the level of knee pain.

**Conclusions:**

Women without knee osteoarthritis tended to report increasing knee pain with increasing age. BMI is considered a significant controllable factor in knee pain in women regardless of the presence of radiographic knee osteoarthritis. The presence of depressive symptoms may aggravate knee pain in women with knee osteoarthritis. Attention needs to be focused on concomitant musculoskeletal problems such as lumbar spinal and hip diseases in women with knee pain.

## Introduction

Controlling knee pain is known to be the most important target of treating knee osteoarthritis, and knee pain is a prognostic factor for radiographic progression of knee osteoarthritis [1]. A previous study reported that knee pain severity was a stronger risk factor for self-reported difficulty in performing physical functions than knee osteoarthritis grade [2]. Therefore, knee pain is a clinically important consideration, both independently and dependently, for knee osteoarthritis.

Previous reports have indicated that knee pain does not necessarily reflect the severity of knee osteoarthritis [3, 4]. The discrepancy between knee pain and radiographic severity of knee osteoarthritis might have been caused by the inclusion of confounding factors and omission of important factors. Moreover, the different study cohorts could have caused conflicting results between studies. Various factors have been known to affect knee pain, including body mass index (BMI), female sex, and depression [5–7].

However, previous studies did not consider the effect of malignant diseases, pain medication or arthritis treatment, or presence of hip or low back pain, which could be confounding factors affecting knee pain. Malignant disease itself and chemotherapy can cause or aggravate arthralgia [8, 9], and pain medication or arthritis treatment can attenuate the corresponding knee pain to a radiographic grade of knee osteoarthritis [10, 11]. Although sufficient evidence is lacking, the presence of hip or spine problems may cause knee pain through either referred pain or increased pain sensitivity in the lower extremity area.

Therefore, the aim was to investigate the factors associated with knee pain in a population-based study, including feasible factors and excluding important confounding factors, such as malignant diseases and osteoarthritis medication, in Korean women aged 50 years and older. We used data from the fifth and sixth Korean National Health and Nutrition Examination Surveys (KNHNES, 2010–2013).

## Methods

### Subjects

Written informed consent was obtained from all participants by the Korean Centers for Disease Control and Prevention, and the study was approved by the Institutional Review Board of Seoul National University Bundang Hospital.

**Fig 1:**
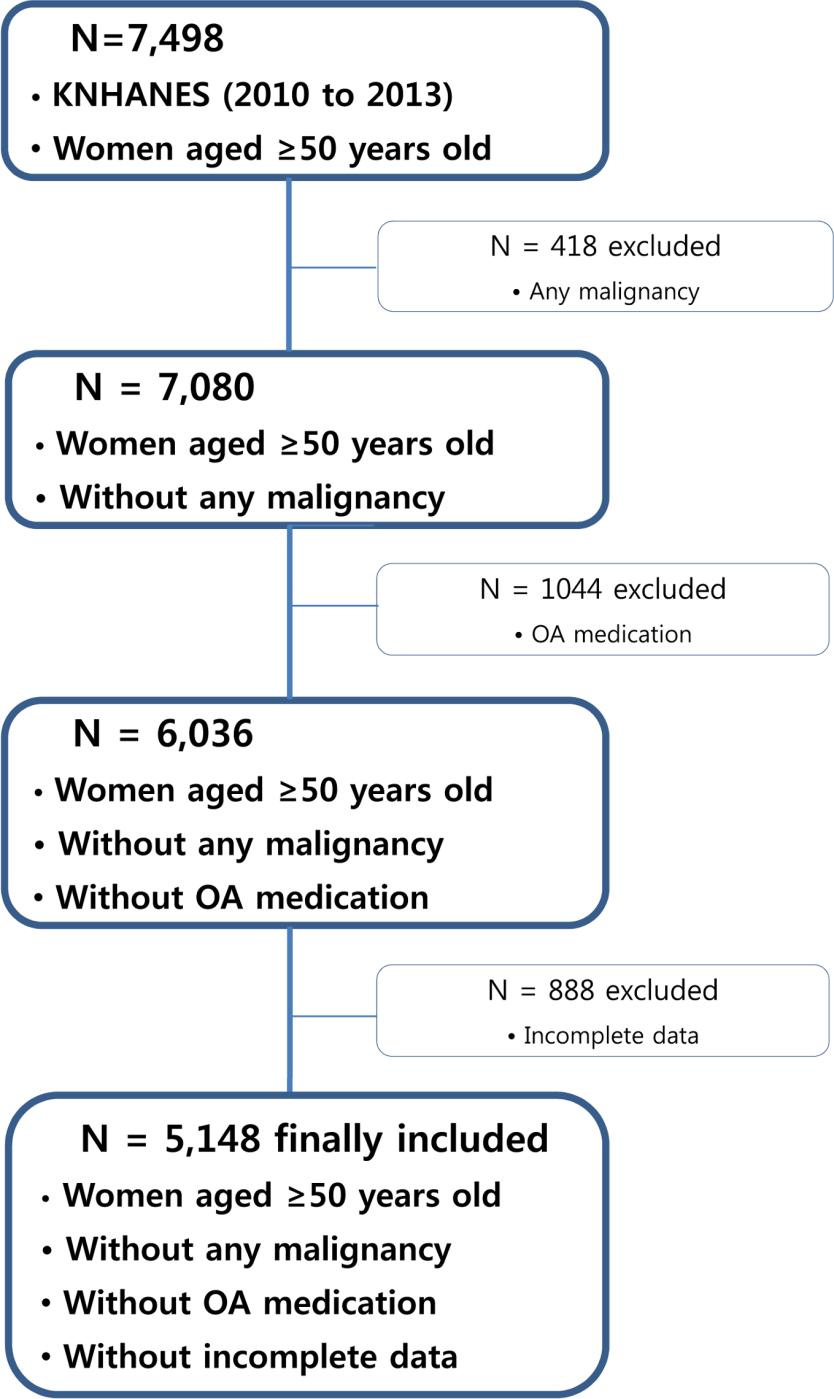
Flow diagram of the inclusion and exclusion criteria for the Korea National Health and Nutrition Examination Survey (KNHNES). A total of 5148 women aged 50 years or older were included.

The KNHNES is a nationally representative cross-sectional database on health and nutrition that is annually conducted by the Korean Centers for Disease Control. A survey of knee osteoarthritis was included in the 2010, 2011, 2012, and 2013 databases. A total of 41,709 subjects were invited to participate in the survey between 2010 and 2013, and 33,551 agreed to participate, corresponding to a response rate of 80.4%. Of these, 7498 women aged 50 years and older were selected. Women who had any malignancy, those who were using osteoarthritis medication, and those with incomplete data were excluded from the data analyses (Fig 1).

### Data collection

Demographic data were collected, including age, sex, BMI, and weight change in the past year (gain of >10, gain of 6–10 kg, gain of 3–6 kg, gain of <3 kg, no change, loss of <3 kg, loss of 3–6 kg, loss of 6–10 kg, and loss of >10 kg). The activity level, using the short form of the International Physical Activity Questionnaire (IPAQ-SF) [12], amount of smoking (current and cumulative), amount of alcohol consumption in the past year, presence of malignant diseases, presence of depressive symptoms, and current use of osteoarthritis medication were recorded.

The survey on knee osteoarthritis and musculoskeletal conditions included knee X-ray imaging and questions about the level of knee and hip pain and presence of low back pain. Pain severity was quantitatively assessed using a 10-point numerical rating scale (NRS). Bilateral weight-bearing anteroposterior knee radiographs were taken using an SD 3000 Synchro Stand (Accele Ray; SYFM Co., Seoul, South Korea) and digitally stored. Radiographic knee osteoarthritis was defined as Kellgren-Lawrence grade 2, 3, or 4 [13]. The radiographic images were reviewed by two radiologists. For the inter-rater reliability test of 81 randomly selected images, the agreement between the two radiologists was 85.2%, with intraclass correlation coefficient of 0.767 (95% confidence interval, 0.659–0.844).

### Data analysis and statistics

The factors associated with the level of knee pain were analyzed for the whole cohort, the subgroup of subjects without radiographic knee osteoarthritis (Kellgren-Lawrence grade 0 or 1), and the subgroup of subjects with radiographic knee osteoarthritis (Kellgren-Lawrence grade 2, 3, or 4).

Descriptive analysis, including the average and standard deviation (SD) or proportion, was performed for all variables. Data normality was tested using the Kolmogorov-Smirnov test. Continuous variables were compared using the Student’s t-test, and categorical variables were compared between the two groups using the chi-square test. The factors significantly associated with the level of knee pain were statistically analyzed using multiple regression analysis and stepwise selection methods. The dependent variable was level of knee pain (NRS), and the independent variables included age, BMI, weight change in the past year, activity level, amount of smoking, amount of alcohol consumption in the past year, presence of depressive symptoms, radiographic grading of knee osteoarthritis (Kellgren-Lawrence grade), level of hip pain, and presence of low back pain. The regression model was built for the whole cohort, the subgroup of subjects without osteoarthritis, and the subgroup of subjects with osteoarthritis.

All statistical analyses were performed using SPSS 20.0 software (IBM Corporation, Armonk, NY), with statistical significance set at p<0.05.

## Results

A total of 5148 women aged 50 years or older were included. The overall prevalence of knee osteoarthritis was 39.4% and that of knee pain was 21.4%. The average age of the subjects was 62.9 years (SD, 9.3 years); 3121 women (average age, 60.0 years; SD, 8.3 years) did not have radiographic knee osteoarthritis and 2027 women (average age, 67.4 years; SD, 9.0 years) had radiographic knee osteoarthritis. Among women without radiographic knee osteoarthritis, 15.0% reported knee pain; among those with radiographic knee osteoarthritis, 31.2% reported knee pain. Age, BMI, level of knee pain, level of hip pain, presence of low back pain, presence of depressive symptom, weight change in the past year, and amount of alcohol consumption in the past year showed significant difference between women without radiographic knee osteoarthritis and those with radiographic knee osteoarthritis (Table 1).

**Table 1:** Data summary.

	Women without knee OA	Women with knee OA	p-value
No.	3121	2027	NA
Age (years)	60.0 (8.3)	67.4 (9.0)	<0.001
BMI (kg/m^2^)	23.6 (3.0)	24.8 (3.4)	<0.001
Knee pain (NRS)	0.8 (2.2)	2.0 (3.3)	<0.001
Kellgren-Lawrence grade (knee joint, 0 / 1 / 2 / 3 / 4)	2046 / 1075 / 0 / 0 / 0	0 / 0 / 771 / 929 / 327	<0.001
Hip pain (NRS)	0.7 (2.1)	0.9 (2.5)	<0.001
Low back pain (yes / no)	2324 / 797	1370 / 657	<0.001
Depressive symptom (yes / no)	2991 / 130	1971 / 56	0.009
Weight change (>10kg gain/6-10kg gain/3-6kg gain/no change/3-6kg loss/ 6-10kg loss/>10kg loss)	23 / 43 / 404 / 2289 / 301 / 47 / 14	13 / 28 / 167 / 1472 / 283 / 53 / 11	<0.001
Current amount of smoking (pieces)	0.4 (2.2)	0.3 (2.0)	0.123
Cumulative amount of smoking (pack)	83.1 (1050.9)	84.5 (911.5)	<0.001
Amount of alcohol consumption (g/day)	1.6 (5.1)	1.1 (4.1)	<0.001
Activity			
Weekly duration of vigorous-intensity activity (min)	73.3 (275.2)	67.0 (329.3)	0.456
Weekly duration of moderate-intensity activity (min)	116.3 (358.5)	123.1 (394.3)	0.524
Weekly duration of walking (min)	241.5 (335.0)	223.7 (350.7)	0.067

Continuous variables are presented as mean (SD). OA, osteoarthritis; NA, not applicable; NRS, numerical rating scale; min, minutes.

For the whole cohort, multiple regression showed that the radiographic grade of knee osteoarthritis, presence of low back pain, level of hip pain, BMI, age, and presence of depressive symptoms were significant and independently associated with the level of knee pain (Table 2).

**Table 2:** Multiple regression analysis to identify significant factors associated with level of knee pain in the whole cohort of women with and without knee OA aged ≥50 years.

	Standardized Beta	t	p-value
Kellgren-Lawrence grade (knee joint, 0 / 1 / 2 / 3 / 4)	0.192	13.416	<0.001
Low back pain (yes / no)	0.208	15.420	<0.001
Hip pain (NRS)	0.216	16.270	<0.001
BMI (kg/m^2^)	0.056	4.337	<0.001
Age (years)	0.058	4.067	<0.001
Depressive symptom (yes / no)	0.041	3.307	0.001
Weight change (>10kg gain/6-10kg gain/3-6kg gain/no change/3-6kg loss/ 6-10kg loss/>10kg loss)	-0.015	-1.136	0.256
Amount of alcohol consumption (g/day)	-0.005	-0.406	0.685
Current amount of smoking (pieces)	0.005	0.425	0.671
Cumulative amount of smoking (pack)	0.017	1.390	0.165
Weekly duration of vigorous-intensity activity (min)	-0.001	-0.085	0.932
Weekly duration of moderate-intensity activity (min)	0.009	0.698	0.485
Weekly duration of walking (min)	0.001	0.110	0.913
Constant	-2.024	-5.347	<0.001

NRS, numerical rating scale; min, minutes. R^2^ = 0.205

For women without knee osteoarthritis, the level of hip pain, presence of low back pain, age, and BMI were found to be significantly and independently associated with the level of knee pain (Table 3).

**Table 3:** Multiple regression analysis to identify significant factors associated with level of knee pain in women without knee osteoarthritis aged ≥50 years.

	Standardized Beta	t	p-value
Hip pain (NRS)	0.266	15.302	<0.001
Low back pain (yes / no)	0.187	10.616	<0.001
Age (years)	0.089	5.291	<0.001
BMI (kg/m^2^)	0.061	3.731	<0.001
Kellgren-Lawrence grade (knee joint, 0 / 1)	0.022	1.315	0.189
Depressive symptom (yes / no)	0.024	1.460	0.144
Weight change (>10kg gain/6-10kg gain/3-6kg gain/no change/3-6kg loss/ 6-10kg loss/>10kg loss)	-0.005	-0.271	0.786
Amount of alcohol consumption (g/day)	0.007	0.437	0.662
Current amount of smoking (pieces)	0.000	-0.009	0.993
Cumulative amount of smoking (pack)	0.024	1.428	0.153
Weekly duration of vigorous-intensity activity (min)	-0.001	-0.032	0.974
Weekly duration of moderate-intensity activity (min)	0.016	0.997	0.319
Weekly duration of walking (min)	-0.007	-0.431	0.666
Constant	-2.047	-5.210	<0.001

NRS, numerical rating scale; min, minutes. R^2^ = 0.164

For women with knee osteoarthritis, the radiographic grade of knee osteoarthritis, presence of low back pain, level of hip pain, presence of depressive symptoms, and BMI were the factors significantly and independently associated with the level of knee pain (Table 4).

**Table 4:** Multiple regression analysis to identify significant factors associated with level of knee pain in women with knee osteoarthritis aged ≥50 years.

	Standardized Beta	t	p-value
Kellgren-Lawrence grade (knee joint, 0 / 1)	0.206	10.117	<0.001
Low back pain (yes / no)	0.246	11.375	<0.001
Hip pain (NRS)	0.178	8.259	<0.001
Depressive symptom (yes / no)	0.073	3.671	<0.001
BMI (kg/m^2^)	0.045	2.228	0.026
Age (years)	0.031	1.411	0.158
Weight change (>10kg gain/6-10kg gain/3-6kg gain/no change/3-6kg loss/ 6-10kg loss/>10kg loss)	-0.026	-1.295	0.195
Amount of alcohol consumption (g/day)	-0.028	-1.424	0.155
Current amount of smoking (pieces)	0.008	0.407	0.684
Cumulative amount of smoking (pack)	0.015	0.743	0.458
Weekly duration of vigorous-intensity activity (min)	-0.012	-0.598	0.550
Weekly duration of moderate-intensity activity (min)	0.000	0.011	0.992
Weekly duration of walking (min)	0.019	0.920	0.358
Constant	-2.584	-4.918	<0.001

NRS, numerical rating scale; min, minutes. R^2^ = 0.193

## Discussion

This study investigated the factors associated with the level of knee pain in community-dwelling women aged 50 years or older. The radiographic grade of knee osteoarthritis (Kellgren-Lawrence grade), presence of low back pain, level of hip pain, BMI, age, and presence of depressive symptoms were significant factors associated with the level of knee pain in the whole cohort. For women without knee osteoarthritis, knee pain was found to increase according to increasing age, BMI, level of hip pain, and presence of low back pain. For women with knee osteoarthritis, knee pain was significantly associated with radiographic grade of knee osteoarthritis, BMI, level of hip pain, presence of low back pain, and presence of depressive symptoms.

Age was an independent risk factor for knee pain after adjustment by radiographic grade of knee osteoarthritis and BMI in the whole cohort. A recent study found that older participants, compared with middle-aged participants, displayed lower mechanical pain thresholds in the quadriceps and epicondyle [14]. Other studies showed that decreased physical activity is associated with increased pain sensitivity in older adults; meanwhile, decreased activity could reduce pain because arthritic pain may be provoked by movement in knee osteoarthritis [15, 16]. Therefore, activity level needs to be considered when evaluating the association between knee pain and age.

In our cohort, age showed a significant negative correlation with weekly hours of vigorous-intensity activity (r = -0.062, p<0.001) and with moderate-intensity activity (r = -0.066, p<0.001), but not with walking activity (r = -0.020, p = 0.157). However, the level of knee pain showed a significant correlation only with weekly hours of walking activity (r = 0.107, p<0.001). In a multiple regression analysis of our data, after adjustment for physical activity, age was a significant factor associated with knee pain. Therefore, older age was significantly associated with knee pain, possibly due to increased pain sensitivity.

It is known that higher BMI is a risk factor for knee osteoarthritis and pain [5, 17]. Overweight increases the mechanical load exerted on the knee joint [18]. In addition, systemic and metabolic factors associated with overweight might play a role in the inflammatory processes caused by adipocytes’ production of inflammatory cytokines [19]. These mechanical and biochemical effects of higher BMI on knee osteoarthritis and knee pain are important clinical factors that could be controlled.

Knee pain was significantly associated with hip pain in women regardless of the presence of knee osteoarthritis in our cohort. Previous studies have indicated that patients with hip disease can present with knee pain [20]. The sensory nerves of both the hip and knee joints originate from the femoral, sciatic, and obturator nerves [21]. Hip joint pathology is known to be an important cause of pain referred to the knee joint. In addition, spinal problems can cause anterior knee pain through radiating pain [22] or through the weakness of the quadriceps muscle. A previous study showed a high percentage of spinal symptoms in patients with knee pain, compared with controls [23]. Although hip or back pain does not necessarily represent hip pathology or radiculopathy, our study results suggest that associated hip and spine disorders need to be evaluated in women with knee pain.

Depressive symptoms were significantly associated with the level of knee pain only in women with knee osteoarthritis. A previous study reported a significant relationship between depression and knee pain in patients with knee osteoarthritis [7], whereas another failed to detect a significant relationship [24]. These conflicting results might have been caused by differences in case numbers and various confounding factors. Our study included a nationally representative sample of women, and we controlled significant confounding factors by excluding women with malignant diseases and those using osteoarthritis medications. The increase in inflammatory cytokines and alteration of neurotransmitter levels caused by depression can affect the level of knee pain because these changes influence the threshold of pain perception [25, 26]. Our study results support the recent use of antidepressants in patients with knee osteoarthritis [27].

This study has some limitations that need to be addressed. First, this was a cross-sectional study, and the causal relationship between knee pain and its associated factors might not be verified by our data. A longitudinal study is required to establish causality of the relevant factors. Second, the effects of the hip and spine problems on knee pain were only evaluated symptomatically. Further objective evidence of hip and spine disorders, such as X-ray images, need to be included in a future study to investigate this issue more comprehensively. Third, this study used simple radiographs to evaluate knee osteoarthritis. However, there are disorders other than osteoarthritis that cause knee pain, such as ligament injuries, meniscal injuries, and osteochondral lesions, especially in younger women; these pathologies could be detected by magnetic resonance imaging. As osteoarthritis is a common knee joint problem in the elderly population, this research setting might have caused an unknown bias in the study. Fourth, our study cohort consisted only of Korean women. Asian cohort is known to be different from the cohort of other ethnic groups. Therefore, our study did not reflect the different ethnic characteristics related to knee pain.
